# 

**Published:** 1903-12-19

**Authors:** 


					/
THE HOSPITAL.  -^f-he Stlfndard of highest purity."?Lancet. December 19th, 1903.
CADBQRV^U^ceU ft* CADBURY'S
COCOA - JL MM Jga COCOA
"A PERFECT FOOD.'r-p ''O- l lra  W NO DRUQS OR ALKALIES UWD.
No. 899. Vol. XXXV. [SfiS5?] Saturday, Dec. 19th, 1903. [Subs^Pp.O202!rms'] PRICE THREEPENCE,

				

